# Detection of specific Chlamydia pneumoniae and cytomegalovirus antigens in human carotid atherosclerotic plaque in a Chinese population

**DOI:** 10.18632/oncotarget.19314

**Published:** 2017-07-18

**Authors:** Jiachao Cao, Yumin Mao, Bo Dong, Wei Guan, Jia Shi, Suinuan Wang

**Affiliations:** ^1^ Department of Neurosurgery, The Third Affiliated Hospital of Soochow University, Changzhou, China

**Keywords:** atherosclerosis, Chlamydia pneumonia, cytomegalovirus, specific antigen, immunohistochemistry detection

## Abstract

To explore the relationship between certain pathogens, such as chlamydia pneumonia (Cpn) and cytomegalovirus (CMV), and carotid atherosclerosis (AS) in a Chinese population.Twenty-five carotid atherosclerotic stenosis patients from the Beijing Tiantan Hospital (affiliated with Capital Medical University) participated in the study. After undergoing digital subtraction angiography (DSA) and/or computed tomography angiography (CTA), the degree of carotid artery stenosis was over 70% in all cases, and the patients underwent carotid endarterectomy. Plaque specimens were obtained during surgery. The streptavidin-peroxidase (SP) method was used to test the Cpn and CMV antigens in the specimens, and the relationship between the Cpn and CMV pathogen infections and AS was analyzed based on the test results. In the group of 25 carotid atherosclerotic specimens, the detection rate of the Cpn-specific antigens was 84.0% (21/25). In the control group, the detection rate was 13.3% (2/15) in the ascending aortic intima. Thus, the between-group difference was significant (P<0.01). The CMV-specific antigen detection rate was 72.0% (18/25) using the same experimental group specimens, and the detection rate was zero in the control group. Thus, there were significant between-group differences (P<0.01). Due to the high detection rate of Cpn- and CMV-specific antigens in carotid atherosclerotic plaque in a Chinese population, it can be inferred that pathogens such as Cpn and CMV are one factor associated with carotid atherosclerosis.

## INTRODUCTION

Atherosclerotic cardiovascular disease has become a leading cause of death in humans. Its traditional risk factors include hyperlipidemia, hypertension, glycuresis, smoking, age, and familial hereditary history [1], but in recent years, increasing evidence has shown that some pathogen infections can promote the occurrence and development of cerebrovascular AS [2][3][4]. The correlation between chlamydia pneumonia (Cpn) and coronary atherosclerotic heart diseases was first reported by Saikku et al. in 1988 [5]. Since then, numerous AS-related pathogens have been discovered. The most common pathogens are Cpn and cytomegalovirus (CMV) [6], but their relationship with As remains unclear. This study discusses the relationship between the Cpn and CMV pathogens and AS by detecting the existence of Cpn and CMV in atheromatous plaque, which was obtained after carotid endarterectomy (CEA) using immunohistochemical methods.

## RESULTS

### The immunohistochemical manifestations of Cpn and CMV

**Figure 1:**
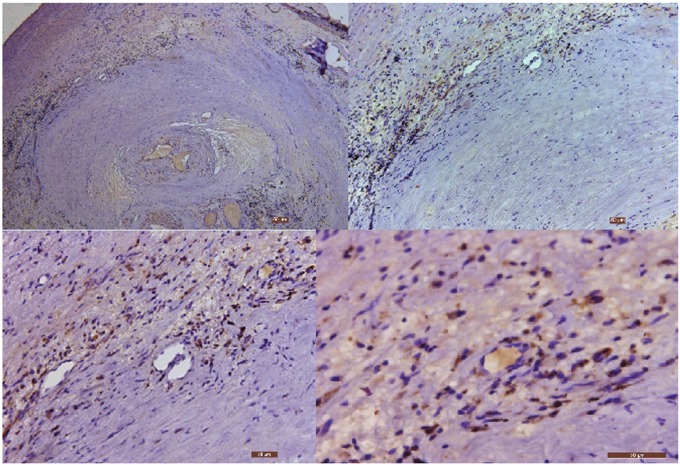
The immunohistochemical staining results of Cpn in carotid atherosclerotic plaque (40×, 100×, 200× and 400×, respectively) Low power microscopy illustrates the serious stenosis of arterial lumen with the formation of blood clots. The nuclei stain is blue, and the granules of Cpn antigens are brown or coffee colored.

**Figure 2:**
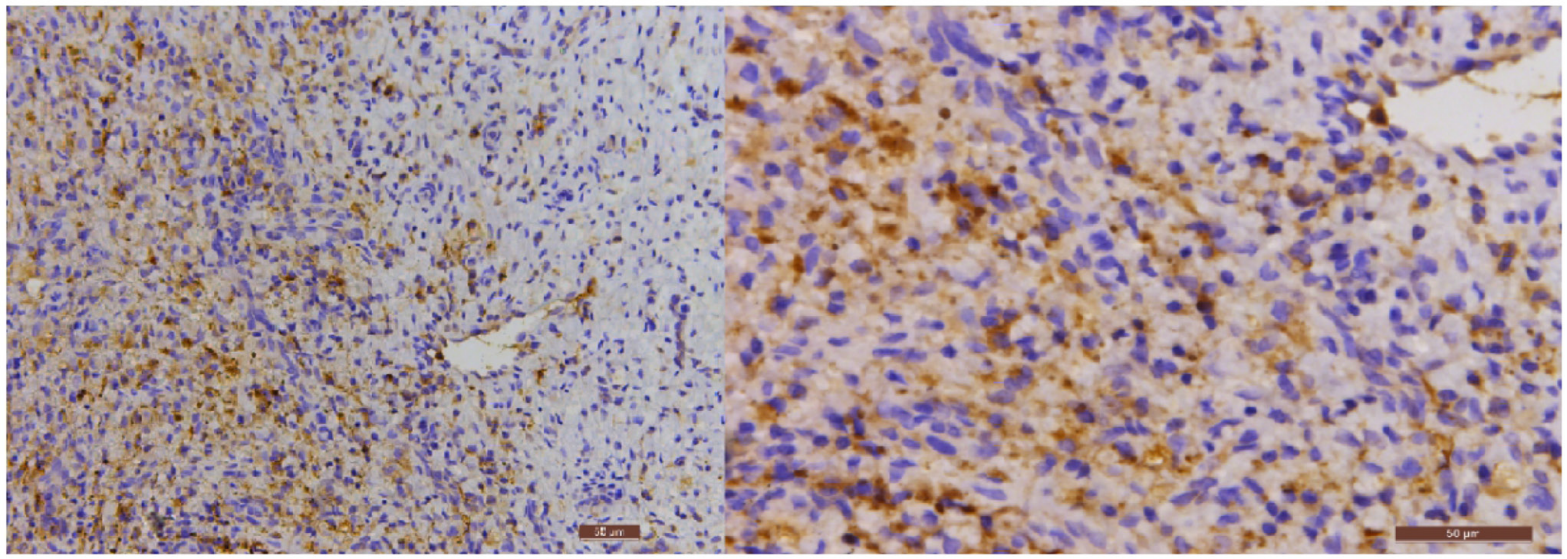
The immunohistochemical staining results of CMV in carotid atherosclerotic plaque (200× and 400×, respectively) The nuclei stain is blue, and the granules of CMV antigens are brown or coffee colored.

The immunohistochemical analysis of Cpn and CMV in the carotid atherosclerotic plaque of the 25 patients in experimental group: the Cpn antigens are shown as brown or coffee granules, and their positive granules are expressed in the cytoplasm and nucleus (Figure 1). ② The location of the positive expression of CMV antigens was similar to that of Cpn, but their positive granules primarily existed in cytoplasm and were expressed in a few nuclei (Figure 2).

### The positive rate has significant differences between the experimental group and the control group

The specific antigens of Cpn in the specimens of the experimental and control group were measured through immunohistochemistry, as shown in Table 1. According to the Chi squared test (x^2^=19.158, P<0.01), the positive rate demonstrated significant differences between the experimental and control groups. The positive rate of Cpn specific antigens in the experimental group was obviously higher than the control group.

**Table 1:** Results of the specific antigens of Cpn in the experimental and control groups specimens, as measured by immunohistochemistry

Group	Number needed to treat	Levels of the specific antigens of Cpn in the specimens measured by immunohistochemistry			
		Strong positive	Positive	Negative	Positive rate(%)
Experimental group	25	2	19	4	84
Control group	15	0	2	13	13.3

Note: x^2^=19.158, P<0.01.

The specific antigens of CMV in the specimens of the experimental and control group were measured through immunohistochemistry, as shown in Table 2. According to the Chi squared test (x^2^=19.636, P<0.01), the positive rate demonstrated significant differences between the experimental and control groups. The positive rate of CMV specific antigens in the experimental group was obviously higher than that in the control group.

**Table 2:** Results of the specific CMV antigens in specimens from the experimental and control groups, as measured by immunohistochemistry

Group	Number needed to treat	Levels of the specific antigens of CMV in the specimens measured using immunohistochemistry			
		Strong positive	Positive	Negative	Positive rate(%)
Experimental group	25	1	17	7	72
Control group	15	0	0	15	0

Note: x^2^=19.636, P<0.01.

### No corresponding relation between the serum antibodies of Cpn and CMV and the immunohistochemical results of carotid atherosclerotic plaque

Cpn IgG and CMV IgG levels in the blood sera of the 25 patients in the experimental group were also tested in this experiment. The antibody positive in the blood serum was compared with the immunohistochemical results to determine whether they have a corresponding relationship (Table 3) (Table 4).

**Table 3:** Comparison of Cpn immunohistochemical test results and serum test results in experimental group

		Levels of the specific antigens of Cpn in the specimens measured using immunohistochemistry		
		Strong positive	Positive	Negative
**Serum**	Positive	1	13	2
**CpnIgG**	Negative	1	6	2

**Table 4:** Comparison of CMV immunohistochemical test results and serum test results in experimental group

		Levels of the specific CMV antigens in the specimens measured using immunohistochemistry		
		Strong positive	Positive	Negative
**Serum**	Positive	1	16	6
**CMVIgG**	Negative	0	1	1

According to the t-test result in Table 5, the Cpn H-score comparative T value of Cpn IgG between negative group and positive group is -0.601, and the corresponding P value is 0.554, which is greater than 0.05 and demonstrates no striking statistical significance, indicating that the index of Cpn H-score has no significant differences in Cpn IgG between negative group and positive group.

**Table 5:** Experimental group Cpn IgG positive and negative immunohistochemical H-score comparison

	Cpn IgG	Sample size (N)	Mean	T value	P value
**Serum**	Negative	9	2.21	-0.601	0.554
**CpnIgG**	Positive	16	2.36		

According to the t-test result shown in Table 6, the CMV H-score comparative T value of CMV IgG between negative group and positive group is -0.241, and the corresponding P value is 0.812, which is greater than 0.05 and demonstrates no striking statistical significance. This result indicates that the index of CMV H-score has no significant differences in CMV IgG between the negative and positive groups.

**Table 6:** Experimental group CMV IgG positive and negative immunohistochemical H-score comparison

	CMV IgG	Sample size (N)	Mean	T value	P value
**Serum**	Negative	2	1.97	-0.241	0.812
**CMVIgG**	Positive	23	2.10		

Based on the above statistical analysis, there is no corresponding relationship between the serum antibodies of Cpn and CMV and the immunohistochemical results of carotid atherosclerotic plaque.

## DISCUSSION

### Atherosclerotic risk factors

AS results from the synergetic action of various risk factors and cannot be accounted for by a single cause. Some traditional risk factors, including hypertension, hypercholesterolemia, and glycuresis [9], have been verified by many studies at home and abroad and are regarded as major inducing factors of AS [5][10]. Although the evidence is circumstantial and the exact action mechanism is still unknown, there is now ample evidence that some pathogen infection is associated with AS, and the strongest evidence is that there are pathogens in lesion vessel walls or atherosclerotic plaques.

### Pathological research on the pathogens and AS

This study is the first to investigate Chinese patients with carotid atherosclerosis. The conventional detection methods used to evaluate the existence status of Cpn and CMV in the same tissue samples include immunohistochemistry, polymerase chain reaction (PCR), and electron microscopes [11][12][13][14]. The immunohistochemical method has higher sensitivity, and the immunohistochemical SP method was used in this study to detect the existence status of Cpn and CMV specific antigens in the carotid atherosclerotic plaque, which was obtained after surgical resection. As shown in Table 1, the detection rate of Cpn specific antigens is 84.0% (21/25) in the 25 carotid atherosclerotic specimens, while it is 13.3% (2/15) in the intima of ascending aorta of the control group. Thus, there are significant differences between the two groups (P<0.01). As shown in Table 2, the detection rate of CMV specific antigens is 72.0% (18/25) for the same specimens in the experimental group, while it is zero in the control group. Thus, there are significant differences between the two groups (P<0.01).

Therefore, it can be inferred that the infection of Cpn and CMV is one possible factor of the formation of AS. There are also some related and similar studies in the literature [15][16] that provide deeper research on the relationship and action mechanism of Cpn infection and the formation of AS. Dniset et al. have argued that the chlamydial lipopolysaccharide (cLPS) and chlamydial heat-shock protein 60 (cHSP60) produced by Cpn play a key role in the process of forming atherosclerotic foam cells, which promote the occurrence and development of AS [17].

The existence of Cpn and CMV specific antibodies in the blood sera of patients in the experimental group was also tested in this study to determine whether there was corresponding relationship with the immunohistochemical results. The testing results are shown in Tables 5 and 6. It was determined that the positive of specific antibodies of pathogens in blood serum cannot indicate the existence status of specific antigens of pathogens in the carotid atherosclerotic plaque; therefore, there is no corresponding relationship between them. The following are possible reasons for this finding. 1. The pathogen was positive in the blood serum but negative in the atheromatous plaque, and in this situation, the major factor of the formation of the atheromatous plaque may have no relationship to Cpn infection. The infection makes the antibody positive in the blood serum, but the pathogen does not invade the artery wall; 2. The pathogen invades the artery wall, and the specific antigens of the pathogen in the carotid atherosclerotic plaque are determined to be positive; therefore, specific antibodies in blood serum do not decline to fail to be detected, or they are repeatedly infected and produce serum antibodies again, which makes the two results consistent. 3. The pathogen localizes on the artery wall for a long time, and the specific antibodies in blood serum decline to fail to be detected; therefore, the pathology result is positive, but serum antibodies are negative. 4. Likely, a few patents with poor immunity cannot produce enough specific antibodies, which also results in further invasion of the artery wall by the pathogen [18].

### Prospective animal experiments investigating the pathogens and AS

In a series of animal experiments, Fong et al. have proven that Cpn has a promotive effect on AS. In the first experiment, four-week old rabbits were intranasally inoculated Cpn, and the control group were administered saline. Thus, the process of human infection was simulated, and all rabbits were fed a normal diet. After two weeks, it was found that the carotid artery of rabbits in the experimental group changed into AS (grade II), while the control group had no correlative changes [19]. In the second experiment, the four-week-old rabbits in the experimental group were intranasally inoculated Cpn in the same manner, and the control group was inoculated with M. Pneumonia. All the rabbits were fed normal diet. After 12 weeks, it was found that the carotid artery of 26.1% (6/23) of the rabbits in the experimental group changed into AS (grade I-III), while the control group still had no correlative changes. The third experiment changed the inoculation to Cpn once every two weeks, and the remaining used the process of the second experiment. After 12 weeks, it was found that the carotid artery of 34.8% (8/23) rabbits in the experimental group changed into AS (grade III-IV), while the control group still had no correlative changes [20]. This result demonstrates that the long-term chronic infection of Cpn can make the carotid artery of rabbits change into AS, and it also partly confirms the results of pathological studies.

Certainly, the experimental conclusion is still worth investigating, as follows. The experiment chooses rabbits as a subject, but if the subject changes to other species, will the results be the same. If the rabbits in the experimental and control groups are fed high fat diets, will the results of the two groups change? If the observation cycle is extended, will the lesion location form typical atherosclerotic plaques and will there be corresponding atherosclerotic changes in the control group? If there plaque forms, will the Cpn markers show positive reactions? These questions require further animal experiments to answer.

Although the high detection rate of Cpn specific antigens in carotid atherosclerotic plaque tissue in a Chinese population cannot establish etiological causality, combined with the results of a series of animal experiments conducted by Fong et al., pathogens such as Cpn and CMV may have a positive effect on the formation of AS and can be risk factors for carotid atherosclerosis. Further studies are still required to confirm the pathogenic causality and mechanism of action and to determine whether there is a cooperative action with other risk factors and whether carotid atherosclerosis patients should undergo anti-infection treatments when they receive optimal internal medicine treatment. It is necessary to design more precise and prospective animal experiments.

## MATERIALS AND METHODS

### Selection of research objectives

Twenty-five AS patients from the Beijing Tiantan Hospital (affiliated with Capital Medical University) participated in the study. After undergoing digital subtraction angiography (DSA) and/or computed tomography angiography (CTA), the degree of carotid artery stenosis was over 70% in all cases, and the patients underwent CEA. In the control group, 15 specimens were taken from parts of the ascending aortas in the coronary atherosclerotic heart disease patients who underwent bypass surgery. In the control group, the selection criterion was no formation of AS at the ascending aorta, according to a low-power field. Statistical information about the clinical features of the research objects is shown in Table 7. The following criteria were used to diagnose hypertension: for patients not using antihypertensive drugs, a systolic pressure greater than or equal to 140 mmHg and a diastolic pressure greater than or equal to 90 mmHg, and patients with past histories of hypertension who were currently using antihypertensive drugs [7]. The following criteria were used to diagnose dyslipidemia: a total cholesterol concentration greater than 200 mg/dl, without considering the serum triglyceride concentration because only elevated levels of triglyceride cannot be considered to be dyslipidemia. Diabetic patients were given insulin during the perioperative period to maintain their blood sugar levels within the normal range. Meanwhile, none of the study participants underwent any antibiotic treatment during the perioperative period [8]. The results shown in Table 7 demonstrate that there was no significant between-group difference in the basic information of patients in the experimental and control groups (P>0.05), which indicates that the patients in the two groups are comparable (Table 7).

**Table 7:** Comparison of patients’ clinical features between the experimental group and the control group

	Experimental group (n=25)	Control group (n=15)	T/x^2^	P^a^
Age^b^	62.96 ± 9.93	64.53 ± 8.58	-0.508	0.614
Male/female	18/7	11/4	0.008	0.927
Smoking	12(48%)	6(40%)	0.242	0.622
history	18(72%)	13(87%)	1.157	0.282
Hypertension	4(16%)	3(20%)	0.104	0.747
Glycuresis Dyslipidemia	10(40%)	4(27%)	0.733	0.392

a: If P<0.05, there is significant difference.

b: Average age ± standard deviation.

### Specimen collection

On the day of surgery (early morning) venous blood samples (3 ml) were collected from each of the 25 carotid artery stenosis patients (after fasting overnight). After anticoagulation and separation of the blood serum, the samples were placed in Eppendorf tubes and conserved in a refrigerator at -80°C for inspection. The plaque specimens were obtained during operation. After washing out blood clots with physiological saline, the samples were immediately fixed in 10% neutral formalin and conserved at 4°C. Then, the samples were made into paraffin blocks through decalcification, dehydration, vitrification and wax dip. The paraffin blocks were conserved in a refrigerator at 4°C for inspection.

The 15 patients in the control group underwent coronary artery bypass graft surgery (CABG). A few ascending aorta specimens were obtained during surgery. They were observed for the formation of AS at a low-power field. Specimens with no AS were included in the control group; specimens with AS were excluded in the control group. After control samples were obtained, they were used as plaque specimens in the experimental group. They were embedded in paraffin blocks and then conserved in a refrigerator at 4°C for inspection.

### Specimen disposal

The enzyme-linked immunosorbent assay (ELISA) was used to test Cpn IgG and CMV IgG in the blood serum of experimental group. ELISA kits were purchased from Abnova, an American company. Meanwhile, the negative control, positive control and quality control were set up, and the indirect method was used to perform the test, according to the manufacturer's specifications. Finally, the patients’ optical density (OD) values were determined through ELISA detectors.

The paraffin-embedded specimens in the experimental and control groups were made into 5 μm sections using routine techniques. The streptavidin-peroxidase method was used to test the Cpn and CMV antigens in the specimens. The specific monoclonal antibodies for Cpn and CMV were purchased from Cellabs, an Australian company, and DaKo, a Danish company, respectively. The negative control was set up for all staining sections, and the control experiment was conducted with phosphate buffer saline (PBS) instead of primary antibodies. The obtained immunohistochemical staining results were determined using a histochemical score (H-score). The staining sections were placed under an Olympus microscopy (CHK model) at high magnification (×400) for observation. Five visual fields were randomly chosen, and each visual field contained 100 cells; the negative (-), weak positive (+), positive (++) and strong positive (+++) cell numbers were determined. For negative cells (-), there were no brown granules in the cytoplasm; for weak positive (+), there were light brown granules in the cytoplasm; for positive (++), there were clear brown granules in the cytoplasm; and for strong positive (+++), there were obvious brown granules in the cytoplasm. The semi-quantitative analysis was conducted using the H-score method. The computational formula is H-score = ∑pi (i + 1), where i refers to staining intensity, and 1, 2 and 3 are used to represent (+), (++) and (+++), respectively; pi represents the percentage of the number of positive cells in the total number of cells to be measured at the same staining intensity, and 1 refers to correction coefficient. The same staining section was determined simultaneously by two researchers, in turn.

### Statistical methods

SSPS software (version 18.0) was used to perform the statistical analysis. The measurement data are presented as the average ± standard deviation, and t-tests were used; Chi squared tests were used for the enumeration data use, and P<0.05 indicated significantly significant differences.

## Abbreviations

Cpn: chlamydia pneumonia
CMV: cytomegalovirus
AS: atherosclerosis
DSA: digital subtraction angiography
CTA: computed tomography angiography
SP: streptavidin-peroxidase
CEA: carotid endarterectomy
CABG: coronary artery bypass graft
b ELISA: enzyme-linked immunosorbent assay
OD: optical density
H-score: histochemical score
PCR: polymerase chain reaction
cLPS: chlamydial lipopolysaccharide
cHSP60: chlamydial heat-shock protein 60
